# Extreme-Temperature Indices and Seasonal Precipitation Deficits Characterize Soybean Yield Variability in Eastern Croatia: The 2024–2025 Climatically Stressful Seasons in Context (2020–2025)

**DOI:** 10.3390/plants15121867

**Published:** 2026-06-16

**Authors:** Tomislav Duvnjak, Aleksandra Sudarić, Anto Mijić, Danijel Jug, Irena Jug, Maja Matoša Kočar, Ana Vuković Vimić, Nina Cvenić

**Affiliations:** 1 Agricultural Institute Osijek, Južno Predgrađe 17, 31000 Osijek, Croatia; aleksandra.sudaric@poljinos.hr (A.S.); anto.mijic@poljinos.hr (A.M.); maja.matosa@poljinos.hr (M.M.K.); nina.cvenic@poljinos.hr (N.C.); 2 Faculty of Agrobiotechnical Sciences Osijek, Josip Juraj Strossmayer University of Osijek, Vladimira Preloga 1, 31000 Osijek, Croatia; djug@fazos.hr (D.J.); ijug@fazos.hr (I.J.); 3 Faculty of Agriculture, University of Belgrade, 6 Nemanjina Street, 11080 Belgrade, Serbia; anavuk@agrif.bg.ac.rs

**Keywords:** soybean, grain yield, hydrothermal stress, heatwaves, tropical nights, drought, maturity group, rainfed agriculture, ANOVA, ANCOVA, climate extremes, Osijek

## Abstract

Recent hydrothermal extremes threaten soybean productivity in rainfed systems of Southeastern Europe, but single-location field datasets require cautious interpretation. This study evaluated cultivar-level soybean yield variability in large-scale rainfed field trials near Osijek, eastern Croatia, during 2020–2025 and compared the climatic context of the adverse 2024 and 2025 seasons. Grain yield, adjusted to 13% moisture, was analyzed across years and maturity groups (MGs) using ANOVA, Fisher’s LSD mean separation, ANCOVA/linear trend analysis, and exploratory climate–yield correlations. Daily station data for 2024–2025 were used to calculate extreme-temperature indices. ANOVA showed a highly significant year effect on yield (*p* < 0.001), whereas MG and Year × MG were not significant. Fisher’s LSD separated 2023 and 2021 as the highest-yielding years, 2024 as intermediate, and 2022 and 2025 as the lowest-yield group. ANCOVA indicated a significant negative common temporal trend (−0.155 t ha^−1^ year^−1^; *p* < 0.001). Yield was positively associated with June–August precipitation (Pearson *r* = 0.885; *n* = 6). The 2024 season showed a stronger heat-related signal, whereas 2025 showed a stronger precipitation-deficit signal. The results should be interpreted within the limits of a site-specific, unbalanced field-trial dataset.

## 1. Introduction

Climate change is increasing the frequency, intensity, and duration of climate extremes, with heat-related hazards and compound events emerging as major risks for agricultural production. Assessment Report of the Intergovernmental Panel on Climate Change (IPCC) identifies temperature- and drought-related climatic impact drivers as key determinants of food-system vulnerability, emphasizing that shifts in extremes may affect crop performance as strongly as changes in mean climate conditions [1]. Consequently, standardized approaches for quantifying climate extremes have become central to climate-impact research and adaptation planning. Widely used frameworks, including those developed by the Expert Team on Climate Change Detection and Indices (ETCCDI), provide harmonized metrics for temperature and precipitation extremes such as hot days, warm nights, and heatwave events [2,3,4]. Recent global assessments further demonstrate that ongoing climate warming is already contributing to measurable yield reductions across major cropping systems [5].

Soybean [(*Glycine max* (L.) Merr.)] is one of the world’s most important crops due to its role as a major source of plant protein and oil. Soybean productivity is strongly influenced by environmental conditions during reproductive development, particularly flowering, pod formation, and seed filling, when heat and water stress can substantially reduce yield [6,7,8]. In Croatia, soybean is a strategically important arable crop, with official statistics reporting approximately 85,000 ha in 2024 and 96,000 ha in 2025 [9]. In eastern Croatian lowland environments around Osijek, soybean production increasingly faces hydrothermal stress during summer, especially under rainfed conditions. Regional studies indicate that water availability can become a major limiting factor, while agronomic practices such as sowing date and stand density also influence yield formation under variable climatic conditions [10,11].

Field and controlled-environment studies consistently show that elevated temperature can substantially reduce soybean yield, particularly when stress coincides with reproductive stages [12,13,14,15]. Heat stress affects photosynthesis, respiration, reproductive success, assimilate partitioning, and seed filling, ultimately limiting yield formation [15,16,17,18]. Soybean reproductive processes are especially vulnerable to high temperatures because elevated heat may reduce pollen viability, pod set, and seed number [19,20,21]. Recent studies additionally emphasize the importance of nighttime heat stress. Warm nights increase respiratory carbon losses, reduce nocturnal recovery, and can negatively affect assimilate utilization during reproductive and seed-filling periods [22,23]. In climate monitoring, “tropical nights” (commonly defined as Tmin ≥ 20 °C) are therefore increasingly used as indicators of nighttime thermal burden [24].

In rainfed systems, soybean yield is also highly sensitive to soil-water availability and the timing of drought relative to phenological development. Earlier studies demonstrated strong stage-dependent responses of soybean to water stress [25], while more recent work confirms that reproductive stages are particularly sensitive to drought-induced reductions in pod set and seed filling [26,27,28,29]. Maturity-group differences may further influence drought sensitivity and “drought escape” through phenological timing [30]. Importantly, a growing body of evidence indicates that combined heat and drought stress can produce stronger yield penalties than either stress alone [31,32,33,34,35]. Such compound stress conditions are increasingly recognized as a major climate-related risk for crop production.

Given the expected increase in climate extremes, soybean adaptation strategies increasingly emphasize climate-risk monitoring, improved stress characterization, and cultivar selection for resilience under heat and drought conditions [36,37,38,39]. Integrating standardized climate-extreme indices with agronomic observations may therefore improve interpretation of stress environments and support adaptation planning in rainfed soybean production systems [40,41,42,43,44,45,46].

Against this background, the present study evaluated soybean yield variability across maturity groups under large-scale rainfed field-trial conditions in eastern Croatia during 2020–2025. Specifically, the analysis (i) quantified interannual yield variation across maturity groups, (ii) tested the effects of year, maturity group, and their interaction using ANOVA and Fisher’s LSD mean separation, (iii) evaluated temporal yield trends using ANCOVA/linear trend analysis, (iv) characterized seasonal temperature and precipitation anomalies relative to the 1991–2020 baseline, (v) compared contrasting hydrothermal stress signals during 2024 and 2025 using daily extreme-temperature indices, and (vi) evaluated winter precipitation accumulation as a potential proxy for pre-season moisture recharge. By combining standardized climate-extreme frameworks with cultivar-level yield observations and formal statistical testing, this study provides information relevant to soybean adaptation and stress-risk interpretation under increasingly variable climatic conditions.

## 2. Results

### 2.1. Interannual Yield Variability, ANOVA Mean Separation and Exploratory Climate–Yield Associations (2020–2025)

**Figure 1:**
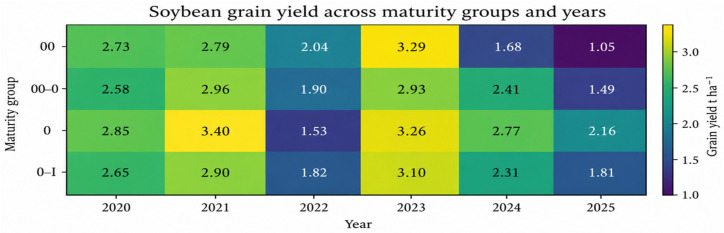
Heatmap of mean soybean grain yield (t ha^−1^) across maturity groups (MG) and years (2020–2025).

Figure 1 summarizes mean grain yield (t ha^−1^) across maturity groups (MGs) and years (2020–2025), highlighting marked interannual variability. Annual mean yield across cultivars varied substantially among years, with the greatest between-cultivar variability observed during the 2024 and 2025 stress seasons (Table 1). Across MGs, yields were highest in 2021 and 2023 and lowest in 2022 and 2025, with 2024 showing intermediate but generally reduced performance compared with the high-yield seasons. Cultivar-level yield observations with three replications per cultivar–year combination where available were analyzed using a general linear model, Fisher’s LSD mean separation and ANCOVA/linear trend analysis.

Formal statistical analysis confirmed a highly significant effect of year on grain yield (*p* < 0.001), whereas the main effect of MG and the Year × MG interaction were not significant at *p* < 0.05 (Table 1b). Fisher’s LSD mean separation showed that 2023 and 2021 had the highest yields, 2020 formed an intermediate high-yield group, 2024 was significantly lower than 2020 but higher than 2022 and 2025, and 2022 and 2025 formed the lowest-yield group (Table 1c). Within individual MGs, the year effect was also evident, although MG-specific results should be interpreted cautiously where the number of cultivars was small (Table 1d). ANCOVA showed no significant Year × MG interaction, indicating that temporal yield trends did not differ significantly among MGs; the common linear trend was negative and significant (−0.155 t ha^−1^ year^−1^; *p* < 0.001; Table 1e). To contextualize this yield variability climatically, Table 1 summarizes annual mean yield together with seasonal temperature and precipitation indicators, while complementary annual-scale climate–yield correlations are provided in Table 1f.

### 2.2. The Year 2024: Season with the Strongest Heat-Related Signal and Concurrent Summer Rainfall Deficit

In 2024, the April–September mean temperature was 21.35 °C, which was +2.77 °C above the 1991–2020 normal (18.58 °C). The summer temperature signal was even stronger: June–August mean temperature reached 25.43 °C, i.e., +3.70 °C above the 1991–2020 normal (21.73 °C). Total April–September rainfall (386.5 mm) was only slightly below the 1991–2020 normal (403.3 mm; −16.8 mm), but the core summer window showed a clear rainfall deficit: June–August rainfall totaled 123.6 mm, corresponding to −89.1 mm relative to the 1991–2020 normal (212.7 mm). Thus, 2024 should not be interpreted as a purely heat-only season; rather, it represented a season with an exceptionally strong heat signal occurring together with a substantial summer rainfall deficit.

As shown in Table 2, the largest descriptive yield reduction in 2024 occurred in MG 00 (−1.03 t ha^−1^; −38.1% relative to the 2020–2023 reference), whereas the other maturity groups showed smaller reductions or values close to the reference yield. In 2025, yield reductions were stronger across all maturity groups, with the largest relative penalty again observed in MG 00 (−1.66 t ha^−1^; −61.3%). These descriptive maturity-group contrasts are consistent with the pattern shown in the MG-specific LSD table, but they should be interpreted within the limits of the unbalanced single-location field-trial dataset and the small number of cultivars in some MG–year combinations.

**Figure 2:**
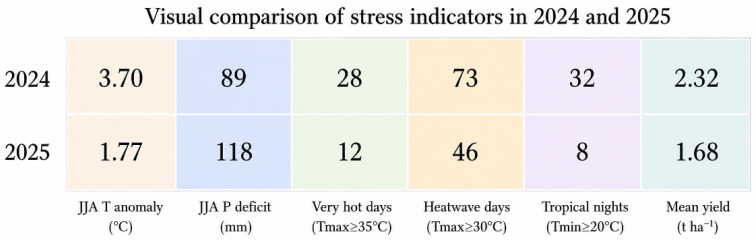
Descriptive comparison of hydrothermal stress indicators in 2024 and 2025, including June–August temperature anomaly, June–August precipitation deficit, very hot days (Tmax ≥ 35 °C), heatwave days (Tmax ≥ 30 °C), tropical nights (Tmin ≥ 20 °C), and annual mean yield. Values are shown in their original units; color intensity is scaled within each indicator only for visual comparison.

Figure 2 summarizes the relative climatic signals of the two adverse seasons. The 2024 season shows the stronger heat-related signal, especially for summer temperature anomaly, very hot days, heatwave days, and tropical nights. The 2025 season shows the stronger precipitation-deficit signal and the lowest mean yield. This comparison is descriptive and does not isolate heat and drought effects statistically.

High-resolution station data for the soybean-relevant period (24 April–1 October) show that 2024 was characterized by pronounced heat exposure: 78 days with Tmax ≥ 30 °C and 28 days with Tmax ≥ 35 °C. Heat stress was also persistent, with 10 heatwave events (≥3 consecutive days with Tmax ≥ 30 °C) totaling 73 heatwave days and a longest run of 17 consecutive hot days (23 Aug.–8 Sep.). Additional evidence of elevated warm-night burden in 2024 is provided by the monthly nighttime-temperature intensity metric (Figure S2). For severe heat (Tmax ≥ 35 °C), 2024 recorded two heatwave events totaling 18 days, including a 10-day severe heatwave (8–17 Jul.). Nocturnal heat stress was substantial, with 32 tropical nights (Tmin ≥ 20 °C), including 12 nights with Tmin ≥ 22 °C and one night with Tmin ≥ 25 °C. Co-occurrence of very hot days and warm nights (Tmax ≥ 35 °C with Tmin ≥ 20 °C) occurred on 20 days, indicating frequent “around-the-clock” thermal stress. In 2024, compound very-hot-day and warm-night events peaked in July and August, whereas in 2025 such events were nearly absent, indicating markedly lower nocturnal heat burden during extreme daytime heat.

Consistent with this combined hot and summer-dry context, mean yield across cultivars in 2024 was 2.32 t ha^−1^, lower than in the higher-yield seasons (2021 and 2023). These results support the interpretation that heat load and warm-night burden were important features of the 2024 stress environment, but they do not allow the relative contribution of heat versus water deficit to be separated statistically.

### 2.3. The Year 2025: Season with the Strongest Precipitation-Deficit Signal and the Lowest Yield

In 2025, April–September mean temperature was 20.08 °C, i.e., +1.50 °C above the 1991–2020 normal (18.58 °C), and June–August mean temperature was 23.50 °C, i.e., +1.77 °C above the normal (21.73 °C). However, the dominant climatic feature of 2025 was precipitation deficit. April–September rainfall totaled only 216.2 mm, representing a large anomaly of −187.1 mm relative to the 1991–2020 normal (403.3 mm). Summer rainfall was also very low: June–August precipitation was 94.6 mm, i.e., −118.1 mm relative to the 1991–2020 normal (212.7 mm).

Compared with 2024, high-resolution station indices indicate lower intensity and persistence of thermal extremes in 2025 (24 April–1 October): 59 days with Tmax ≥ 30 °C and 12 days with Tmax ≥ 35 °C. Heatwave activity for Tmax ≥ 30 °C comprised nine events totaling 46 heatwave days, with the longest run lasting 10 consecutive days (8–17 Aug.). For Tmax ≥ 35 °C, 2025 had one heatwave event meeting the ≥3-day definition (3 consecutive days, 14–16 Aug.). Nocturnal heat stress was much weaker than in 2024, with eight tropical nights (Tmin ≥ 20 °C) and no nights with Tmin ≥ 22 °C; co-occurrence of Tmax ≥ 35 °C and Tmin ≥ 20 °C occurred on only one day.

**Figure 3:**
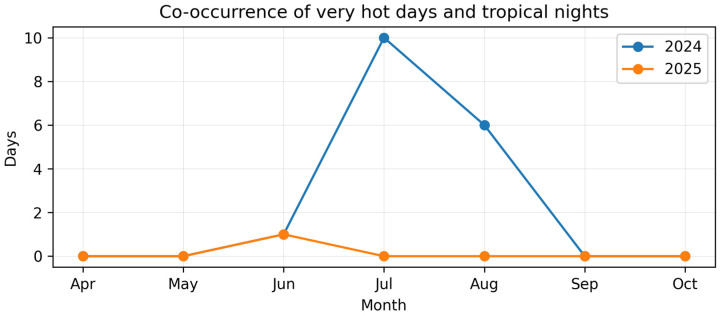
Comparison of monthly counts of days combining very hot daytime temperatures and tropical nights (Tmax ≥ 35 °C and Tmin ≥ 20 °C) during the soybean growing window (24 April–1 October), 2024–2025.

Because co-occurrence days (Tmax ≥ 35 °C with Tmin ≥ 20 °C) represent a particularly severe ‘around-the-clock’ heat load, their monthly distribution is shown in Figure 3.

Despite fewer extreme-heat and warm-night events than in 2024, 2025 produced the lowest mean yield across cultivars (1.68 t ha^−1^). This pattern is consistent with the very large precipitation deficit across both the broader growing-season window (April–September) and the summer critical period (June–August), suggesting that water limitation was an important climatic constraint in 2025, with heat acting as an additional stressor. Because soil-moisture measurements were not available, this interpretation is based on precipitation anomalies and should be viewed as a climatic proxy for water limitation rather than a direct measurement of plant-available water.

### 2.4. Comparative Interpretation of 2024 and 2025 Relative to the 1991–2020 Baseline

Both 2024 and 2025 departed strongly from the 1991–2020 baseline, but the dominant climatic signals differed. The 2024 season showed the stronger heat-related signal, particularly through summer temperature anomaly, heatwave persistence, tropical nights, and co-occurrence of very hot days and warm nights; however, it also included a clear summer rainfall deficit. The 2025 season showed the stronger precipitation-deficit signal across both April–September and June–August, while thermal extremes and warm-night frequency were lower than in 2024. Therefore, the terms heat-related and precipitation-deficit-related are used here as relative descriptors of the dominant climatic signals in the two seasons, not as evidence of isolated single-factor causation.

### 2.5. Winter Precipitation Accumulation as a Proxy for Pre-Season Moisture Recharge

**Figure 4:**
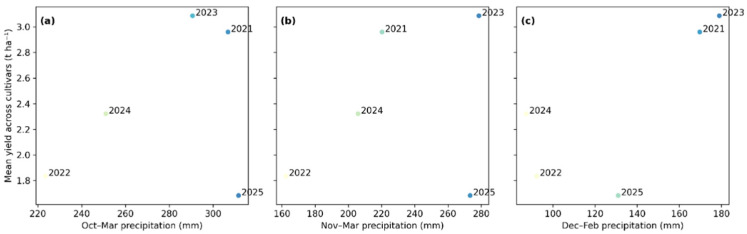
Relationship between mean soybean yield across cultivars (t ha^−1^) and winter precipitation totals (mm) for 2021–2025, calculated for (**a**) October–March, (**b**) November–March, and (**c**) December–February. Points are labeled by year; winter precipitation is used as a proxy for potential pre-season soil-water recharge. Correlation results are interpreted descriptively because of the limited number of years (*n* = 5).

To examine whether pre-season precipitation could partly explain interannual yield variation under rainfed conditions, mean yield across cultivars (2021–2025) was related to winter precipitation totals calculated for alternative accumulation windows (Oct.–Mar., Nov.–Mar. and Dec.–Feb.). The relationships between mean soybean yield and winter precipitation totals calculated for the three accumulation windows are shown in Figure 4.

Winter precipitation totals and associated seasonal indicators are summarized in Table S1, while Pearson and Spearman correlations between mean yield and winter precipitation across the three accumulation windows are reported in Table S2. Across windows, the association was weak and inconsistent, and 2025 emerged as a clear outlier, combining relatively high winter precipitation with the lowest yield. This indicates that winter precipitation alone was not a reliable stand-alone indicator of yield outcome in this dataset, particularly when severe in-season precipitation deficits occurred.

## 3. Discussion

### 3.1. Scope of Inference and Interannual Yield Variability

Soybean yield under large-scale, rainfed field-trial conditions near Osijek varied markedly during 2020–2025. The cultivar-level dataset with three replications per cultivar–year combination where available allowed formal testing of year, maturity group and Year × MG effects, while the single-location and unbalanced nature of the dataset still limits broader regional generalization. The ANOVA indicated that year was the dominant source of yield variability, whereas MG and the Year × MG interaction were not significant at *p* < 0.05. These results indicate that interannual climatic and production conditions explained a larger share of yield variability than maturity-group classification in this dataset.

Fisher’s LSD mean separation clarified the ranking of years: 2023 and 2021 had the highest yields, 2020 was intermediate, 2024 was significantly lower than 2020 but higher than 2022 and 2025, and 2022 and 2025 formed the lowest-yield group. The ANCOVA/linear trend analysis further indicated a significant negative common temporal trend across 2020–2025, although this trend should be interpreted cautiously because it covers only six years and is strongly influenced by the climatically adverse 2024 and 2025 seasons. Complementary climate–yield correlations supported the agronomic importance of summer water supply, with June–August precipitation showing the strongest positive association with annual mean yield.

### 3.2. Relative Climatic Signals in 2024 and 2025

The contrast between 2024 and 2025 is best interpreted as a difference in relative climatic signal rather than a strict separation between heat and drought. The 2024 season showed the strongest heat-related signal, with the highest summer temperature anomaly, frequent hot days, many tropical nights, and repeated co-occurrence of very hot days and warm nights. At the same time, summer rainfall was also below the 1991–2020 normal, indicating that heat effects likely occurred together with some degree of water limitation.

In 2025, the main climatic feature was the exceptionally large precipitation deficit across both April–September and June–August. Thermal extremes were less intense and nocturnal heat burden was much lower than in 2024, yet yield reached its minimum. This pattern supports the interpretation that in-season precipitation deficit was the dominant climatic signal in 2025, although the absence of soil-moisture measurements prevents direct quantification of plant-available water stress.

### 3.3. Maturity-Group Response and Management Implications

Earlier maturity groups, particularly MG 00 and MG 00–0, showed larger descriptive yield penalties in the adverse seasons, but the overall ANOVA did not detect a significant MG effect or Year × MG interaction at *p* < 0.05. This indicates that maturity-group patterns should be considered agronomically relevant but statistically cautious in this single-location, unbalanced dataset. The observed pattern remains plausible because maturity group and phenological timing influence the overlap between sensitive reproductive stages and periods of maximum heat or water limitation. Nevertheless, because detailed phenological observations were not available, the study cannot determine the exact growth stages affected by individual stress events. Future analyses should link daily extreme indices to cultivar-specific flowering, pod-setting and seed-filling dates.

From an applied perspective, the results suggest that cultivar deployment and sowing-window decisions should consider both summer precipitation risk and the increasing likelihood of high-temperature and warm-night events. The findings also support the value of monitoring tropical nights and co-occurrence of very hot days with warm nights, because these indices may capture stress environments not adequately represented by mean seasonal temperature alone.

### 3.4. Limitations and Future Work

Several limitations must be considered. First, the dataset comes from a single location, so broader regional generalization requires multi-location validation. Second, although cultivar-level yield observations with three replications were available, the dataset was unbalanced because not all cultivars were present in all years. Third, daily extreme-temperature indices were available only for 2024–2025, whereas the 2020–2023 seasons were characterized using seasonal monthly summaries. Fourth, soil-moisture, pest-pressure, disease-pressure and soil-fertility measurements were not available, so their interactions with heat and precipitation deficits could not be quantified. Finally, seasonal climate windows were fixed calendar periods rather than phenology-specific exposure windows. These limitations have been made explicit to align the interpretation with the strength of the available data.

Future work should extend the dataset across locations and years, maintain balanced cultivar representation where possible, and link daily climatic extremes to phenological stages and soil-water measurements. Such data would allow more robust testing of heat–drought interactions and maturity-group differences under the rainfed production conditions of eastern Croatia.

## 4. Materials and Methods

### 4.1. Study Area and Site Conditions

The study was conducted in Osijek (eastern Croatia), on production fields located in the vicinity of the Agricultural Institute Osijek (45.540675° N, 18.736017° E). The environment is representative of local rainfed soybean production. The soil is classified as Eutric Cambisol. The effective exploitable soil profile was approximately 40 cm, referring to the main soil layer considered relevant for crop water availability in this production setting; no direct soil-water retention measurements were available for the present analysis. Soybean was cultivated under non-irrigated conditions. Crop rotation was typical for the region, with soybean commonly grown after winter wheat, barley or maize, depending on the year.

### 4.2. Field Scale and Experimental Design

Yield data were obtained from single-location, large-scale field trials conducted under production conditions in the same local area around the Institute. Fields were production units of approximately 10 ha or larger. Each cultivar–year combination available in the dataset included three grain-yield replications. Because not all cultivars were included in all years, the dataset represented an unbalanced cultivar-level field-trial dataset. This supports applied relevance for commercial rainfed production, while still requiring site-specific interpretation rather than broad multi-location generalization. Standard agronomic management practices for the region were applied throughout the study period (e.g., conventional soil preparation, fertilization and crop protection as required), and no supplemental irrigation was used.

### 4.3. Plant Material and Yield Data

The dataset comprised soybean cultivars developed within the breeding program of the Agricultural Institute Osijek (Department of Industrial Plants Breeding and Genetics) and classified into maturity groups (MG: 00, 00–0, 0 and 0–I). Annual grain yield was compiled for the period 2020–2025. For each year, the mean yield was calculated (i) across all cultivars (overall annual mean) and (ii) within each maturity group (MG-specific annual mean), where MG means represent the average across cultivars belonging to the respective MG in a given year. Annual mean yield values are presented as mean ± standard deviation (SD). Grain yield was adjusted to a standard grain moisture content of 13% before statistical analysis.

### 4.4. Sowing and Harvest Windows

Sowing and harvest occurred within the typical regional windows, with year-specific ranges as follows, as shown in Table 3.

### 4.5. Climate Data Sources and Baseline Normal

Temperature and precipitation data (monthly summaries for 2020–2025 and daily records for 2024–2025) were provided by the Croatian Meteorological and Hydrological Service (DHMZ; Državni hidrometeorološki zavod, Croatia) upon formal request for scientific use, using the nearest official DHMZ station to the study fields (Klisa, Osijek). Climate normals for 1991–2020 supplied with the dataset were used as the primary reference baseline to calculate temperature and precipitation anomalies. The dataset also included long-term averages for additional reference periods (1961–1990 and 1961–2010), which were retained for descriptive context.

### 4.6. Definition of Seasonal Windows

For seasonal characterization, climate indicators were summarized for:

- April–September, representing the main soybean growing-season window;
- June–August, representing the core summer period critical for water availability and thermal stress.

For extreme-temperature analysis using high-resolution station records, the soybean-relevant period was defined as late April to 1 October, consistent with data availability and covering the main vegetative and reproductive phases under local conditions.

### 4.7. Climate Indicators and Anomalies

Monthly mean air temperature (Tmean, °C) and precipitation totals (P, mm) were aggregated to seasonal values. Temperature anomalies (ΔT) and precipitation anomalies (ΔP) were computed as departures from the 1991–2020 baseline:

ΔT = Tmean (season, year) − Tmean (season, 1991–2020),

ΔP = P (season, year) − P (season, 1991–2020).

### 4.8. Extreme-Temperature Indices (2024–2025)

To quantify heat exposure during the soybean-relevant period (late April–1 October) in 2024 and 2025, daily maximum and minimum temperature records from a local station were used to compute threshold-based indices.

Daily indices were computed for the soybean-relevant period (late April–1 October) using local station Tmax and Tmin records: hot days (HD30; Tmax ≥ 30 °C), very hot days (VHD35; Tmax ≥ 35 °C), heatwave days (HWD30; days belonging to events of ≥3 consecutive days with Tmax ≥ 30 °C, including the longest event duration), tropical nights (TN20; Tmin ≥ 20 °C), and co-occurrence days (Tmax ≥ 35 °C with Tmin ≥ 20 °C).

### 4.9. Winter Precipitation Accumulation (Pre-Season Moisture Proxy)

To assess potential pre-season soil-water recharge, winter precipitation totals were calculated for three alternative accumulation windows: ONDJFM (October–March), NDJFM (November–March), and DJF (December–February). Winter totals were reported as absolute precipitation (mm) and as percentages relative to the corresponding 1991–2020 normal for each window.

### 4.10. Data Processing and Statistical Analysis

All calculations were performed using cultivar-level yield data organized by year, cultivar, maturity group (MG) and replication. For each cultivar–year combination where the cultivar was present, three replicated yield observations were available. For descriptive summaries, cultivar yields were averaged within MGs to obtain MG means, and an overall annual mean yield across cultivars was computed to support year-level climate–yield comparisons. A 2020–2023 reference was calculated as the MG mean yield averaged across 2020–2023; yield deviations for 2024 and 2025 were then expressed as absolute differences (Δ; t ha^−1^) and relative differences (%).

Seasonal climate indicators were summarized for April–September and June–August, including mean air temperature and precipitation totals, and expressed both as absolute values and as anomalies relative to the 1991–2020 normal for the corresponding period. For the high-resolution station dataset (2024–2025), daily extreme-temperature indices were calculated for the soybean-relevant window (24 April–1 October), including hot days (Tmax ≥ 30 °C), very hot days (Tmax ≥ 35 °C), tropical nights (Tmin ≥ 20 °C) and co-occurrence days (Tmax ≥ 35 °C with Tmin ≥ 20 °C). Heatwave events were defined as periods of ≥3 consecutive days with Tmax ≥ 30 °C; both the number of events and the total number of heatwave days were reported. Winter precipitation accumulation was quantified as total precipitation for ONDJFM (Oct.–Mar.), NDJFM (Nov.–Mar.), and DJF (Dec.–Feb.) and expressed as mm and as a percentage of the 1991–2020 normal.

A general linear model was used to test the effects of year, maturity group (MG), and their interaction on grain yield. Where significant effects were detected, Fisher’s LSD test was used for mean separation at *p* < 0.05. Mean-separation tables were generated for year effects, overall MG effects, and year means within each MG. Because the dataset was unbalanced, results were interpreted within the context of a single-location cultivar-level field-trial dataset.

In addition, an ANCOVA/linear trend model was fitted with year treated as a continuous covariate and MG as a categorical factor and the Year × MG interaction was included to test whether temporal slopes differed among maturity groups. Associations between annual mean yield and selected climate indicators were explored using Pearson product–moment and Spearman rank correlation coefficients. Correlations were calculated at the annual scale (*n* = 6 for 2020–2025 seasonal indicators; *n* = 5 for winter precipitation analyses, 2021–2025). Statistical summaries, ANOVA, LSD mean separation, ANCOVA and exploratory correlations were calculated using IBM SPSS Statistics, version 27.0 (IBM Corp., Armonk, NY, USA) and cross-checked in Python, version 3.13.5. Summary tables were prepared in Microsoft Excel for Microsoft 365 (Microsoft Corp., Redmond, WA, USA), and figures were regenerated at high resolution to improve readability.

## 5. Conclusions

Under rainfed, large-scale field-trial conditions in eastern Croatia, soybean grain yield varied considerably among years during 2020–2025. Formal ANOVA confirmed a highly significant year effect on yield, while the main effect of maturity group and the Year × MG interaction were not significant at *p* < 0.05. Fisher’s LSD mean separation identified 2023 and 2021 as the highest-yielding years, 2024 as an intermediate reduced-yield year, and 2022 and 2025 as the lowest-yield group. ANCOVA further indicated a significant negative common temporal trend across 2020–2025, although this trend should be interpreted cautiously because of the short time span and the influence of climatically adverse seasons. The 2024 season showed the strongest heat-related signal, including an exceptional summer temperature anomaly, frequent heatwaves and a high warm-night burden, but it also included a clear summer rainfall deficit. The 2025 season showed the strongest precipitation-deficit signal across both April–September and June–August and had the lowest mean yield, despite fewer thermal extremes than 2024. Overall, the study supports the practical value of combining formal yield statistics, seasonal precipitation anomalies, heatwave metrics, tropical nights and yield observations to characterize stress environments in rainfed soybean production. The findings should be interpreted within the limits of a single-location, unbalanced field-trial dataset and should be validated with multi-location, multi-year datasets including soil moisture and phenological observations.

## Figures and Tables

**Table 1:** (**a**) Annual soybean yield and seasonal climate indicators relative to the 1991–2020 baseline in the Osijek area during 2020–2025. (**b**) Two-way ANOVA for soybean grain yield using cultivar-level observations with three replications per cultivar–year combination where available. (**c**) Fisher’s LSD mean comparison for year and maturity-group (MG) effects on soybean grain yield. (**d**) Fisher’s LSD comparison of year means within each maturity group (MG). (**e**) ANCOVA/linear trend analysis of soybean grain yield with year treated as a continuous covariate and maturity group (MG) as a categorical factor. (**f**) Complementary exploratory correlations between annual mean soybean yield across cultivars and seasonal climate indicators during 2020–2025.

(a)							
Year		2020	2021	2022	2023	2024	2025
Mean yield across cultivars ± SD (t ha^−1^)		2.67 ± 0.42	2.96 ± 0.49	1.84 ± 0.30	3.09 ± 0.47	2.32 ± 0.71	1.68 ± 0.59
Tmean April–September (°C)		18.70	18.85	19.73	19.63	21.35	20.08
Tmean April–September anomaly vs. 1991–2020 (°C)		0.12	0.27	1.15	1.04	2.77	1.50
P April–September (mm)		320.4	333.5	268.6	362.1	386.5	216.2
P April–September anomaly vs. 1991–2020 (mm)		−82.9	−69.8	−134.7	−41.2	−16.8	−187.1
Tmean June–August (°C)		21.97	23.37	23.90	22.95	25.43	23.50
Tmean June–August anomaly vs. 1991–2020 (°C)		0.23	1.63	2.17	1.22	3.70	1.77
P June–August (mm)		211.0	194.1	66.5	178.7	123.6	94.6
P June–August anomaly vs. 1991–2020 (mm)		−1.7	−18.6	−146.2	−34.0	−89.1	−118.1
(b)							
Source		Df	SS	MS	F		*p*-value
Year		5	52.816	10.563	43.23		<0.001
MG		3	1.630	0.543	2.22		0.088
Year × MG		15	4.524	0.302	1.23		0.251
Error		159	38.848	0.244			
(c)							
Factor		Level	*n*	Mean Yield (t ha^−1^)	SE		LSD Group
Year		2023	33	3.089	0.081		a
Year		2021	24	2.961	0.096		a
Year		2020	27	2.669	0.078		b
Year		2024	33	2.322	0.120		c
Year		2022	33	1.835	0.051		d
Year		2025	33	1.684	0.100		d
MG		0	18	2.666	0.156		a
0MG		0–I	99	2.397	0.079		b
MG		00–0	48	2.329	0.087		b
MG		00	18	2.264	0.184		b
(d)							
MG	Year	*n*	Mean Yield (t ha^−1^)		SE		LSD Group
00	2020	3	2.730		0.015		ac
00	2021	3	2.793		0.024		ac
00	2022	3	2.040		0.038		bc
00	2023	3	3.287		0.155		a
00	2024	3	1.680		0.051		bd
00	2025	3	1.053		0.055		d
00–0	2020	6	2.578		0.151		ac
00–0	2021	6	2.958		0.054		a
00–0	2022	9	1.897		0.056		b
00–0	2023	9	2.937		0.085		a
00–0	2024	9	2.409		0.127		c
00–0	2025	9	1.489		0.049		b
0	2020	3	2.863		0.023		ac
0	2021	3	3.400		0.021		a
0	2022	3	1.533		0.028		b
0	2023	3	3.263		0.018		a
0	2024	3	2.773		0.033		ac
0	2025	3	2.160		0.057		bc
0–I	2020	15	2.655		0.128		d
0–I	2021	12	2.895		0.180		ad
0–I	2022	18	1.821		0.083		c
0–I	2023	18	3.103		0.138		a
0–I	2024	18	2.311		0.200		b
0–I	2025	18	1.807		0.161		c
(e)							
Effect		Df	SS	MS	F		*p*-Value
Year numeric × MG		3	1.473	0.491	1.05		0.374
Year numeric (common slope)		1	12.375	12.375	26.35		<0.001
MG adjusted for trend		3	1.652	0.551	1.17		0.322
Error (reduced model)		178	83.608	0.470			
Common slope (t ha^−1^ year^−1^)		—	−0.155	SE = 0.030	t = −5.13		<0.001
(f)							
Indicator		Pearson *r*	Pearson *p*	Spearman *p*	Spearman *p*		*n*
Tmean April–September		−0.437	0.386	−0.600	0.208		6
P April–September		0.744	0.090	0.657	0.156		6
Tmean June–August		−0.374	0.465	−0.600	0.208		6
P June–August		0.885	0.019	0.714	0.111		6

Note: (a) Yield values represent annual mean yield across cultivars grown under rainfed conditions (mean ± SD). Climate anomalies are expressed relative to the 1991–2020 reference period. Climate indicators are interpreted as contextual variables because detailed daily extreme-temperature indices were available only for 2024–2025. (b) Sequential sums of squares are reported; the residual mean square from the full model was used for Fisher’s LSD mean separation. (c) Means sharing at least one letter are not significantly different at *p* < 0.05. (d) Letter groupings were calculated using the residual mean square from the two-way ANOVA; MG-specific results should be interpreted cautiously where the number of cultivars is small. (e) Year was coded as a continuous covariate (2020 = 0, …, 2025 = 5). Because the Year numeric × MG interaction was not significant, the common slope from the reduced model is reported. (f) Correlations are calculated at the annual scale using *n* = 6 years. They are reported as complementary effect-size indicators only; *p*-values are shown to document uncertainty and should be interpreted cautiously because of the small sample size.

**Table 2:** Relative yield changes in the climatically stressful seasons 2024 and 2025 compared with the 2020–2023 maturity-group reference means.

MG	Reference Yield 2020–2023 (t ha^−1^)	Yield 2024 (t ha^−1^)	Yield 2025 (t ha^−1^)	Δ 2024 (t ha^−1^)	Δ 2025 (t ha^−1^)	Relative Yield Change 2024 (%)	Relative Yield Change 2025 (%)
0	2.76	2.77	2.16	0.00	−0.60	0.1	−21.6
0–I	2.62	2.31	1.81	−0.30	−0.81	−11.6	−31.0
00	2.71	1.68	1.05	−1.03	−1.66	−38.1	−61.3
00–0	2.59	2.41	1.49	−0.18	−1.10	−7.0	−42.4

Note: Reference values correspond to the mean maturity-group yield during 2020–2023 under rainfed conditions. Deviations for 2024 and 2025 are presented for descriptive comparison with the pre-stress reference period; formal ANOVA and LSD mean-separation results are presented in Table 1b–d.

**Table 3:** Sowing and harvest windows for soybean large-scale production fields in the Osijek area during 2020–2025.

Year	Sowing		Harvest	
	from	to	from	to
2020	7 April	7 May	11 September	22 September
2021	12 April	8 May	13 September	5 October
2022	13 April	4 May	6 September	15 September
2023	14 April	5 May	11 September	19 September
2024	16 April	10 May	26 August	28 September
2025	24 April	17 May	17 September	4 October

## Data Availability

The data presented in this study are available on request from the corresponding author due to third-party restrictions on the redistribution of meteorological data obtained from the Croatian Meteorological and Hydrological Service (DHMZ) under a formal request for scientific use.

## Supplementary Materials

The following supporting information can be downloaded at: https://www.mdpi.com/article/10.3390/plants15121867/s1, Table S1a—Annual mean soybean yield and seasonal climate indicators relative to the 1991–2020 baseline; Table S1b—Winter precipitation totals across alternative accumulation windows and annual mean soybean yield during 2021–2025; Table S1c—Daily extreme-temperature indices for 2024–2025; Table S2—Pearson and Spearman correlations between annual mean soybean yield and winter precipitation totals calculated for alternative accumulation windows during 2021–2025; Table S3—Cultivar-level grain yield dataset used for analysis of variance (ANOVA), Fisher’s least significant difference (LSD) mean comparison, and analysis of covariance (ANCOVA); Table S4a—Two-way ANOVA for soybean grain yield; Table S4b—Fisher’s LSD mean comparison for years; Table S4c—Fisher’s LSD mean comparison for maturity groups; Table S4d—Fisher’s LSD mean comparison for year means within each maturity group; Table S5a—ANCOVA/linear trend analysis; Table S5b—Common linear trend estimate; Figure S1—Relationship between annual mean soybean yield across cultivars (t ha^−1^) and June–August (JJA) precipitation total (mm) for 2020–2025; Figure S2—Monthly number of warm nights in 2024 and 2025 during the soybean-relevant period (24 April–1 October), defined as nights when daily minimum temperature exceeded the monthly 90th-percentile threshold calculated from station records for the 2024–2025 reference period.

## References

[1] IPCC (2021). Annex VI: Climatic impact-driver and extreme indices. Climate Change 2021: The Physical Science Basis.

[2] Klein Tank A.M.G., Zwiers F.W. (2009). Guidelines on Analysis of Extremes in a Changing Climate in Support of Informed Decisions for Adaptation.

[3] Expert Team on Climate Change Detection and Indices (ETCCDI) (2009). Climate Change Indices: Definitions of the 27 Core Indices. https://etccdi.pacificclimate.org/list_27_indices.shtml.

[4] European Climate Assessment & Dataset (ECA&D) (2016). Indices of Daily Temperature and Precipitation Extremes (ETCCDI/ETCCDM Index Comparison). https://www.ecad.eu/dailydata/index.php.

[5] Lobell D.B., Schlenker W., Costa-Roberts J. (2011). Climate trends and global crop production since 1980. Science.

[6] Egli D.B., Singh G. (2010). Soybean yield physiology: Principles and processes of yield production. The Soybean: Botany, Production and Uses.

[7] Tacarindua C.R.P., Shiraiwa T., Homma K., Kumagai E., Sameshima R. (2012). The response of soybean seed growth characteristics to increased temperature under near-field conditions in a temperature gradient chamber. Field Crops Res..

[8] Vogel J.T., Liu W., Olhoft P., Crafts-Brandner S.J., Pennycooke J.C., Christiansen N. (2021). Soybean Yield Formation Physiology—A Foundation for Precision Breeding Based Improvement. Front. Plant Sci..

[9] Croatian Bureau of Statistics (2024). First Estimate of Areas of Some Important Crops, 2024.

[10] Pospišil A., Pospišil M. (2024). Prinos i komponente prinosa soje u optimalnom i naknadnom roku sjetve. Poljoprivreda.

[11] Galić Subašić D., Rapčan I., Jurišić M., Petrović D., Radočaj D. (2024). The Effect of Irrigation on the Yield and Soybean (*Glycine max* L. Merr.) Seed Germination in the Three Climatically Varying Years. Poljoprivreda.

[12] Siebers M.H., Yendrek C.R., Drag D., Locke A.M., Rios Acosta L., Leakey A.D.B., Ainsworth E.A., Bernacchi C.J., Ort D.R. (2015). Heat waves imposed during early pod development in soybean cause significant yield loss despite a rapid recovery from oxidative stress. Glob. Change Biol..

[13] Thomey M.L., Slattery R.A., Köhler I.H., Bernacchi C.J., Ort D.R. (2019). Yield response of field-grown soybean exposed to heat waves under current and elevated [CO_2_]. Glob. Change Biol..

[14] Ruiz-Vera U.M., Siebers M., Gray S.B., Drag D.W., Rosenthal D.M., Kimball B.A., Ort D.R., Bernacchi C.J. (2013). Global warming can negate the expected CO_2_ stimulation in photosynthesis and productivity for soybean grown in the Midwestern United States. Plant Physiol..

[15] Poudel S., Adhikari B., Dhillon J., Reddy K.R., Stetina S.R., Bheemanahalli R. (2023). Quantifying the Physiological, Yield, and Quality Plasticity of Southern USA Soybeans under Heat Stress. Plant Stress.

[16] Sana A., Shahani A.A.A., Ihsan U., Hameed R., Abbas A., Balooch S., Summiya F., Zulfiqar U., Prasad P.V.V., Djalovic I. (2025). Traversing the heat—A review on heat stress untangling the modern approaches in soybean (*Glycine max* (L.)). Plant Stress.

[17] Guan J., Gai Y., Guan Y., Rasheed A., Zhao Q., Xie Z., Mahmood A., Zhang S., Zhang Z., Zhao Z. (2022). Improvement of heat stress tolerance in soybean (*Glycine max* L.), by using conventional and molecular tools. Front. Plant Sci..

[18] Kalantar Ahmadi S.A., Daneshian J. (2025). Enhancing Soybean (*Glycine max* L. Merr) Heat Stress Tolerance: Effects of Sowing Date on Seed Yield, Oil Content, and Fatty Acid Composition in Hot Climate Conditions. Food Sci. Nutr..

[19] Van der Laan L., de Azevedo Peixoto L., Singh A.K. (2025). Genetic dissection of heat stress tolerance in soybean through genome-wide association studies and use of genomic prediction to enhance breeding applications. npj Sci. Plants.

[20] Chebrolu K.K., Fritschi F.B., Ye S., Krishnan H.B., Smith J.R., Gillman J.D. (2016). Impact of heat stress during seed development on soybean seed metabolome. Metabolomics.

[21] Feng Z., Ding C.Q., Li W.H., Wang D.C., Cui D. (2020). Applications of metabolomics in the research of soybean plant under abiotic stress. Food Chem..

[22] Djanaguiraman M., Prasad P.V.V., Boyle D.L., Schapaugh W.T. (2013). Soybean pollen anatomy, viability and pod set under high temperature stress. J. Agron. Crop Sci..

[23] Djanaguiraman M., Prasad P.V.V., Schapaugh W.T. (2013). High day- or nighttime temperature alters leaf assimilation, reproductive success, and phosphatidic acid of pollen grain in soybean (*Glycine max* (L.) Merr.). Crop Sci..

[24] Walker L.P. (2012). Screening Soybean Lines for Heat-Tolerant Reproductive Traits. Master’s Thesis.

[25] Yang L., Song W., Xu C., Sapey E., Jiang D., Wu C. (2023). Effects of high night temperature on soybean yield and compositions. Front. Plant Sci..

[26] Sankarapillai L.V., Adhikari B., Bista M.K., Shrestha A., Stetina S.R., Reddy K.R., Bheemanahalli R. (2025). High night temperature disrupts the assimilate utilization and yield potential in soybean. Plant Stress.

[27] European Environment Agency (EEA) (2026). Tropical Nights Indicator (Metadata/Definition), Climate-ADAPT. https://climate-adapt.eea.europa.eu/en/metadata/indicators/tropical-nights.

[28] Eck H.V., Mathers A.C., Musick J.T. (1987). Plant water stress at various growth stages and growth and yield of soybeans. Field Crops Res..

[29] Du Y., Zhao Q., Chen L., Yao X., Zhang H., Wu J., Xie F. (2020). Effect of Drought Stress during Soybean R2–R6 Growth Stages on Sucrose Metabolism in Leaf and Seed. Int. J. Mol. Sci..

[30] Rasheed A., Mahmood A., Maqbool R., Albaqami M., Sher A., Sattar A., Bakhsh G., Nawaz M., Hassan M.U., Al-Yahyai R. (2022). Key insights to develop drought-resilient soybean: A review. J. King Saud. Univ. Sci..

[31] Yerzhebayeva R., Didorenko S., Bastaubayeva S., Amangeldiyeva A., Maikotov B., Kassenov R., Shavrukov Y. (2024). Soybean Drought Tolerance and Escape: Field Trial Assessment of Yield, Maturity Groups and Smooth-Wrinkled Seed Coats in Kazakhstan. Agriculture.

[32] Zhou Q., Song S., Wang X., Yan C., Ma C., Dong S. (2022). Effects of drought stress on flowering soybean physiology under different soil conditions. Plant Soil Environ..

[33] Wang C., Sun A., Zhu L.J., Liu M., Zhang Q., Wang L., Gao X. (2025). Drought and rewatering effects on soybean photosynthesis, physiology and yield. PeerJ.

[34] Wijewardana C., Reddy K.R., Alsajri F.A., Irby J.T., Krutz J., Golden B. (2018). Quantifying soil moisture deficit effects on soybean yield and yield component distribution patterns. Irrig. Sci..

[35] Wijewardana C., Reddy K.R., Krutz L.J., Gao W., Bellaloui N. (2019). Drought stress has transgenerational effects on soybean seed germination and seedling vigor. PLoS ONE.

[36] Sintaha M., Man C.-K., Yung W.-S., Duan S., Li M.-W., Lam H.-M. (2022). Drought Stress Priming Improved the Drought Tolerance of Soybean. Plants.

[37] Cohen I., Zandalinas S.I., Huck C., Fritschi F.B., Mittler R. (2021). Meta-analysis of drought and heat stress combination impact on crop yield and yield components. Physiol. Plant..

[38] Cohen I., Zandalinas S.I., Fritschi F.B., Sengupta S., Fichman Y., Azad R.K., Mittler R. (2021). The impact of water deficit and heat stress combination on the molecular response, physiology, and seed production of soybean. Physiol. Plant..

[39] Jumrani K., Bhatia V.S., Pandey G.P. (2018). Impact of combined stress of high temperature and water deficit on growth and seed yield of soybean. Physiol. Mol. Biol. Plants.

[40] Poudel S., Vennam R.R., Sankarapillai L.V., Liu J., Reddy K.R., Wijewardane N.K., Mukhtar M.S., Bheemanahalli R. (2024). Negative synergistic effects of drought and heat during flowering and seed setting in soybean. Environ. Exp. Bot..

[41] Ergo V.V., Lascano H.R., Vega C.R.C., Parola R., Carrera C.S. (2018). Heat and water stressed field-grown soybean: A multivariate study on the relationship between physiological-biochemical traits and yield. Environ. Exp. Bot..

[42] Abdelhakim L.O.A., Zhou R., Ottosen C.-O. (2022). Physiological Responses of Plants to Combined Drought and Heat under Elevated CO_2_: A Review. Agronomy.

[43] Bigolin T., Talamini E. (2025). Impacts of Climate Change on Late Soybean Cultivation in Subtropical Southern Brazil. Crops.

[44] Islam M.R., Abdullah H.M., Rahman M.F., Islam M., Tuhin A.K., Ashiquzzaman M., Islam K.S., Geisseler D. (2025). Mitigation of Water-Deficit Stress in Soybean by Seaweed Extract: The Integrated Approaches of UAV-Based Remote Sensing and a Field Trial. Drones.

[45] Li M., Liu Y., Pan Y., Zhang X., Xu K., Qu Y., Li H. (2024). Quantifying High-Temperature and Drought Stress Effects on Soybean Growth and Yield in the Western Guanzhong Plain. Atmosphere.

[46] Zhang L., Yu Q., Yin X., Liu L., Ren Z., Fang Z., Shen W., Liu S., Liu B. (2025). Changes in the Stress Response and Fitness of Hybrids Between Transgenic Soybean and Wild-Type Plants Under Heat Stress. Plants.

