# Supplementary material for: Extreme-Temperature Indices and Seasonal Precipitation Deficits Characterize Soybean Yield Variability in Eastern Croatia: The 2024–2025 Climatically Stressful Seasons in Context (2020–2025)
Source: Plants (Basel). 2026 Jun 16;15(12):1867. doi: 10.3390/plants15121867 (PMC13307521; doi:10.3390/plants15121867)

Supplementary Materials

Extreme-Temperature Indices and Seasonal Precipitation Deficits Characterize Soybean Yield Variability in Eastern Croatia: The 2024–2025 Climatically Stressful Seasons in Context (2020–2025)

Tomislav Duvnjak et al.

The following supporting information is provided:

Table S1a—Annual mean soybean yield and seasonal climate indicators relative to the 1991–2020 baseline;

Table S1b—Winter precipitation totals across alternative accumulation windows and annual mean soybean yield during 2021–2025;

Table S1c—Daily extreme-temperature indices for 2024–2025;

Table S2—Pearson and Spearman correlations between annual mean soybean yield and winter precipitation totals calculated for alternative accumulation windows during 2021–2025;

Table S3—Cultivar-level grain yield dataset used for analysis of variance (ANOVA), Fisher's least significant difference (LSD) mean comparison, and analysis of covariance (ANCOVA);

Table S4a—Two-way ANOVA for soybean grain yield;

Table S4b—Fisher's LSD mean comparison for years;

Table S4c—Fisher's LSD mean comparison for maturity groups;

Table S4d—Fisher's LSD mean comparison for year means within each maturity group;

Table S5a—ANCOVA/linear trend analysis;

Table S5b—Common linear trend estimate;

Figure S1—Relationship between annual mean soybean yield across cultivars (t ha^{-1}) and June–August precipitation total (mm) during 2020–2025;

Figure S2—Monthly number of warm nights in 2024 and 2025 during the soybean-relevant period (24 April–1 October), defined as nights when daily minimum temperature exceeded the monthly 90th-percentile threshold calculated from station records for the 2024–2025 reference period.

The cultivar-level grain yield data are presented by year, cultivar, maturity group, and replication. Yield values are expressed as t ha^{-1} and were adjusted to a standard grain moisture content of 13%. The dataset represents a single-location, large-scale field-trial dataset obtained under rainfed production conditions. Statistical results should therefore be interpreted within this site-specific and unbalanced experimental context.

Table S1a. Annual mean soybean yield and seasonal climate indicators relative to the 1991–2020 baseline.

Year	Mean yield (t ha⁻¹)	SD	Fisher LSD group (Year)	Tmean Apr–Sep (°C)	Tmean Apr–Sep anomaly vs 1991–2020 (°C)	P Apr–Sep (mm)	P Apr–Sep anomaly vs 1991–2020 (mm)	Tmean Jun–Aug (°C)	Tmean Jun–Aug anomaly vs 1991–2020 (°C)	P Jun–Aug (mm)	P Jun–Aug anomaly vs 1991–2020 (mm)
2020	2.67	0.42	b	18.7	0.12	320.4	-82.9	21.97	0.23	211	-1.7
2021	2.96	0.49	a	18.85	0.27	333.5	-69.8	23.37	1.63	194.1	-18.6
2022	1.84	0.3	d	19.73	1.15	268.6	-134.7	23.9	2.17	66.5	-146.2
2023	3.09	0.47	a	19.63	1.04	362.1	-41.2	22.95	1.22	178.7	-34
2024	2.32	0.71	c	21.35	2.77	386.5	-16.8	25.43	3.7	123.6	-89.1
2025	1.68	0.59	d	20.08	1.5	216.2	-187.1	23.5	1.77	94.6	-118.1

Table S1b. Winter precipitation totals across alternative accumulation windows and annual mean soybean yield (2021–2025).

Year	Mean yield (t ha⁻¹)	Oct–Mar precipitation (mm)	Nov–Mar precipitation (mm)	Dec–Feb precipitation (mm)
2021	2.96	306.8	220.7	168.0
2022	1.84	223.4	162.0	92.5
2023	3.09	290.7	279.6	176.9
2024	2.32	251.1	206.1	87.8
2025	1.68	311.5	272.2	130.6

Note: Winter precipitation totals were calculated for the alternative accumulation windows used in the manuscript.

Table S1c. Daily extreme-temperature indices for 2024–2025.

Year	Hot days Tmax ≥30 °C (2024–2025 only)	Very hot days Tmax ≥35 °C (2024–2025 only)	Heatwave events Tmax ≥30 °C	Heatwave days Tmax ≥30 °C	Longest hot-day run (days)	Tropical nights Tmin ≥20 °C	Co-occurrence days Tmax ≥35 °C and Tmin ≥20 °C
2024	78	28	10	73	17	32	20
2025	59	12	9	46	10	8	1

Table S2. Pearson and Spearman correlations between annual mean soybean yield and winter precipitation totals calculated for alternative accumulation windows (2021–2025).

Winter precipitation window	Pearson *r*	Pearson *p*	Spearman *ρ*	Spearman *p*	*n*
Oct–Mar	0.343	0.572	-0.100	0.873	5
Nov–Mar	0.309	0.613	0.400	0.505	5
Dec–Feb	0.738	0.155	0.600	0.285	5

Note: Correlations are exploratory and should be interpreted cautiously because only five years were available.

Table S3. Cultivar-level grain yield dataset used for ANOVA, Fisher's LSD mean comparison, and ANCOVA.

Cultivar	MG	Year	Replication	Yield (t ha^{-1})
V1	00	2025	I	0.95
V1	00	2025	II	1.14
V1	00	2025	III	1.07
V1	00	2024	I	1.78
V1	00	2024	II	1.61
V1	00	2024	III	1.65
V1	00	2023	I	3.36
V1	00	2023	II	2.99
V1	00	2023	III	3.51
V1	00	2022	I	1.97
V1	00	2022	II	2.1
V1	00	2022	III	2.05
V1	00	2021	I	2.76
V1	00	2021	II	2.78
V1	00	2021	III	2.84
V1	00	2020	I	2.7
V1	00	2020	II	2.75
V1	00	2020	III	2.74
V2	00–0	2025	I	1.76
V2	00–0	2025	II	1.44
V2	00–0	2025	III	1.5
V2	00–0	2024	I	2.93
V2	00–0	2024	II	2.88
V2	00–0	2024	III	2.84
V2	00–0	2023	I	3.12
V2	00–0	2023	II	3.09
V2	00–0	2023	III	3.17
V2	00–0	2022	I	2.1
V2	00–0	2022	II	2.16
V2	00–0	2022	III	2.07
V3	00–0	2025	I	1.34
V3	00–0	2025	II	1.48
V3	00–0	2025	III	1.37
V3	00–0	2024	I	2.25
V3	00–0	2024	II	2.38
V3	00–0	2024	III	2.32
V3	00–0	2023	I	3.06
V3	00–0	2023	II	3.15
V3	00–0	2023	III	3.03
V3	00–0	2022	I	1.77
V3	00–0	2022	II	1.82
V3	00–0	2022	III	1.87
V3	00–0	2021	I	3.02
V3	00–0	2021	II	3.08

Cultivar	MG	Year	Replication	Yield (t ha^{-1})
V3	00–0	2021	III	3.12
V3	00–0	2020	I	2.25
V3	00–0	2020	II	2.28
V3	00–0	2020	III	2.2
V4	00–0	2025	I	1.56
V4	00–0	2025	II	1.64
V4	00–0	2025	III	1.31
V4	00–0	2024	I	2.04
V4	00–0	2024	II	2.07
V4	00–0	2024	III	1.97
V4	00–0	2023	I	2.65
V4	00–0	2023	II	2.57
V4	00–0	2023	III	2.59
V4	00–0	2022	I	1.8
V4	00–0	2022	II	1.78
V4	00–0	2022	III	1.7
V4	00–0	2021	I	2.85
V4	00–0	2021	II	2.81
V4	00–0	2021	III	2.87
V4	00–0	2020	I	2.87
V4	00–0	2020	II	2.92
V4	00–0	2020	III	2.95
V5	0	2025	I	2.08
V5	0	2025	II	2.27
V5	0	2025	III	2.13
V5	0	2024	I	2.74
V5	0	2024	II	2.74
V5	0	2024	III	2.84
V5	0	2023	I	3.29
V5	0	2023	II	3.27
V5	0	2023	III	3.23
V5	0	2022	I	1.59
V5	0	2022	II	1.5
V5	0	2022	III	1.51
V5	0	2021	I	3.37
V5	0	2021	II	3.39
V5	0	2021	III	3.44
V5	0	2020	I	2.9
V5	0	2020	II	2.87
V5	0	2020	III	2.82
V6	0–I	2025	I	1.44
V6	0–I	2025	II	1.28
V6	0–I	2025	III	1.35
V6	0–I	2024	I	2.69
V6	0–I	2024	II	2.66

Cultivar	MG	Year	Replication	Yield (t ha⁻¹)
V6	0–I	2024	III	2.56
V6	0–I	2023	I	3.93
V6	0–I	2023	II	3.83
V6	0–I	2023	III	3.89
V6	0–I	2022	I	1.6
V6	0–I	2022	II	1.52
V6	0–I	2022	III	1.51
V6	0–I	2020	I	2.21
V6	0–I	2020	II	2.27
V6	0–I	2020	III	2.14
V7	0–I	2025	I	1.31
V7	0–I	2025	II	1.27
V7	0–I	2025	III	1.24
V7	0–I	2024	I	3.15
V7	0–I	2024	II	3.2
V7	0–I	2024	III	3.19
V7	0–I	2023	I	2.39
V7	0–I	2023	II	2.45
V7	0–I	2023	III	2.37
V7	0–I	2022	I	2.04
V7	0–I	2022	II	2.11
V7	0–I	2022	III	2.12
V7	0–I	2021	I	3.83
V7	0–I	2021	II	3.92
V7	0–I	2021	III	3.86
V7	0–I	2020	I	3.01
V7	0–I	2020	II	3.02
V7	0–I	2020	III	3.07
V8	0–I	2025	I	1.32
V8	0–I	2025	II	1.37
V8	0–I	2025	III	1.29
V8	0–I	2024	I	2.36
V8	0–I	2024	II	2.41
V8	0–I	2024	III	2.3
V8	0–I	2023	I	3.15
V8	0–I	2023	II	3.08
V8	0–I	2023	III	3.2
V8	0–I	2022	I	1.79
V8	0–I	2022	II	1.88
V8	0–I	2022	III	1.78
V9	0–I	2025	I	1.79
V9	0–I	2025	II	1.84
V9	0–I	2025	III	1.73
V9	0–I	2024	I	0.57
V9	0–I	2024	II	0.54

Cultivar	MG	Year	Replication	Yield (t ha⁻¹)
V9	0–I	2024	III	0.62
V9	0–I	2023	I	2.84
V9	0–I	2023	II	2.77
V9	0–I	2023	III	2.74
V9	0–I	2022	I	1.27
V9	0–I	2022	II	1.36
V9	0–I	2022	III	1.31
V9	0–I	2021	I	2.77
V9	0–I	2021	II	2.76
V9	0–I	2021	III	2.7
V9	0–I	2020	I	2.51
V9	0–I	2020	II	2.49
V9	0–I	2020	III	2.45
V10	0–I	2025	I	2.05
V10	0–I	2025	II	1.86
V10	0–I	2025	III	1.83
V10	0–I	2024	I	2.41
V10	0–I	2024	II	2.37
V10	0–I	2024	III	2.34
V10	0–I	2023	I	2.64
V10	0–I	2023	II	2.63
V10	0–I	2023	III	2.56
V10	0–I	2022	I	2.41
V10	0–I	2022	II	2.36
V10	0–I	2022	III	2.31
V10	0–I	2021	I	2.25
V10	0–I	2021	II	2.3
V10	0–I	2021	III	2.21
V10	0–I	2020	I	2.11
V10	0–I	2020	II	2.22
V10	0–I	2020	III	2.19
V11	0–I	2025	I	3.22
V11	0–I	2025	II	3.05
V11	0–I	2025	III	3.28
V11	0–I	2024	I	2.75
V11	0–I	2024	II	2.7
V11	0–I	2024	III	2.77
V11	0–I	2023	I	3.84
V11	0–I	2023	II	3.8
V11	0–I	2023	III	3.74
V11	0–I	2022	I	1.77
V11	0–I	2022	II	1.85
V11	0–I	2022	III	1.79
V11	0–I	2021	I	2.68
V11	0–I	2021	II	2.75

Cultivar	MG	Year	Replication	Yield (t ha^{-1})
V11	0–I	2021	III	2.71
V11	0–I	2020	I	3.37
V11	0–I	2020	II	3.52
V11	0–I	2020	III	3.24

Note: Yield values are expressed as t ha^{-1} and adjusted to 13% grain moisture. Replications I–III were used as plot-level observations.

Table S4a. Two-way ANOVA for grain yield: Yield = Year + MG + Year × MG.

Source	df	SS	MS	F	*p*-value
Year	5	52.816	10.563	43.23	<0.001
MG	3	1.63	0.543	2.22	0.088
Year × MG	15	4.524	0.302	1.23	0.251
Error	159	38.848	0.244		
Total	182	97.817			

Table S4b. Fisher's LSD mean comparison for years.

Year	*n*	Mean yield (t ha^{-1})	SD	SE	LSD group
2020	27	2.669	0.407	0.078	b
2021	24	2.961	0.47	0.096	a
2022	33	1.835	0.296	0.051	d
2023	33	3.089	0.464	0.081	a
2024	33	2.322	0.691	0.120	c
2025	33	1.684	0.577	0.100	d

Table S4c. Fisher's LSD mean comparison for maturity groups.

MG	*n*	Mean yield (t ha^{-1})	SD	SE	LSD group
0	18	2.666	0.664	0.156	a
0–I	99	2.397	0.79	0.079	b
00–0	48	2.329	0.601	0.087	b
00	18	2.264	0.782	0.184	b

Table S4d. Fisher's LSD mean comparison for year means within each maturity group.

MG	Year	*n*	Mean yield (t ha^{-1})	SD	SE	LSD group
00	2020	3	2.73	0.026	0.015	ac
00	2021	3	2.793	0.042	0.024	ac
00	2022	3	2.04	0.066	0.038	bc
00	2023	3	3.287	0.268	0.155	a
00	2024	3	1.68	0.089	0.051	bd
00	2025	3	1.053	0.096	0.055	d
00–0	2020	6	2.578	0.369	0.151	ac
00–0	2021	6	2.958	0.131	0.054	a
00–0	2022	9	1.897	0.168	0.056	b
00–0	2023	9	2.937	0.254	0.085	a
00–0	2024	9	2.409	0.38	0.127	c
00–0	2025	9	1.489	0.147	0.049	b
0	2020	3	2.863	0.04	0.023	ac
0	2021	3	3.4	0.036	0.021	a
0	2022	3	1.533	0.049	0.028	b
0	2023	3	3.263	0.031	0.018	a
0	2024	3	2.773	0.058	0.033	ac
0	2025	3	2.16	0.098	0.057	bc
0–I	2020	15	2.655	0.496	0.128	d
0–I	2021	12	2.895	0.623	0.18	ad

MG	Year	*n*	Mean yield (t ha^{-1})	SD	SE	LSD group
0–I	2022	18	1.821	0.353	0.083	c
0–I	2023	18	3.103	0.584	0.138	a
0–I	2024	18	2.311	0.847	0.2	b
0–I	2025	18	1.807	0.684	0.161	c

Table S5a. ANCOVA/linear trend analysis.

Effect	df	SS	MS	F	*p*-value
Year_numeric × MG	3	1.473	0.491	1.05	0.374
Year_numeric (common slope)	1	12.375	12.375	26.35	<0.001
MG adjusted for trend	3	1.652	0.551	1.17	0.322
Error (reduced model)	178	83.608	0.47		

Table S5b. Common linear trend estimate.

Parameter	Estimate	SE	*t*	*p*-value	95% CI lower	95% CI upper
Common linear trend (t ha^{-1} $year^{-1}$)	−0.155	0.030	−5.13	<0.001	−0.215	−0.096

Figure S1. Relationship between annual mean soybean yield across cultivars (t ha⁻¹) and June–August (JJA) precipitation total (mm) for 2020–2025. Points are labeled by year.

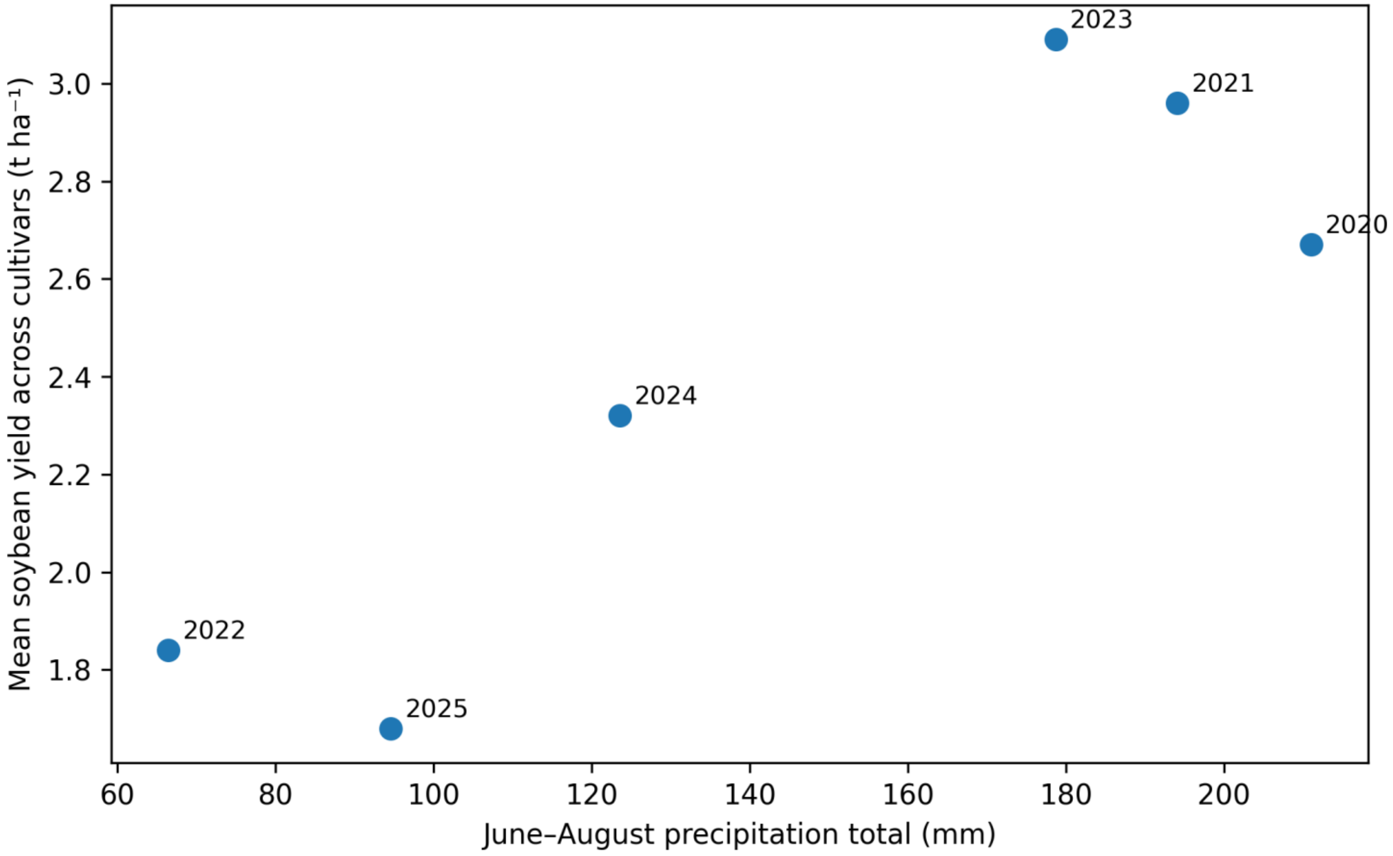

Figure S2. Monthly number of warm nights in 2024 and 2025 during the soybean-relevant period (24 April–1 October), defined as nights when daily minimum temperature (Tmin) exceeded the monthly 90th-percentile threshold calculated from station records for the 2024–2025 reference period. Higher values indicate a greater frequency of warm nights during the growing season.

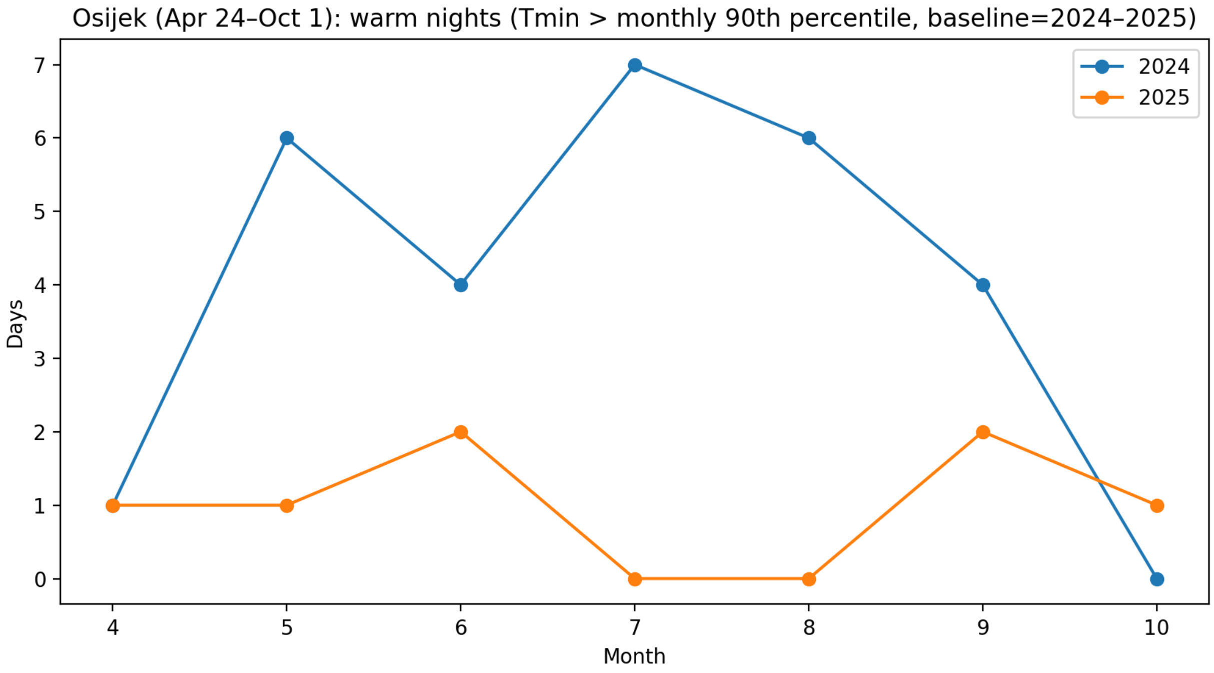

Supplement: Supplementary file 1 [file plants-15-01867-s001.zip › plants-4340683-supplementary.pdf]
